# The effect of peak serum estradiol level during ovarian stimulation on cumulative live birth and obstetric outcomes in freeze-all cycles

**DOI:** 10.3389/fendo.2023.1130211

**Published:** 2023-07-17

**Authors:** Jiaan Huang, Yao Lu, Yaqiong He, Yuan Wang, Qinling Zhu, Jia Qi, Ying Ding, Hanting Zhao, Ziyin Ding, Yun Sun

**Affiliations:** ^1^ Center for Reproductive Medicine, Ren Ji Hospital, School of Medicine, Shanghai Jiao Tong University, Shanghai, China; ^2^ Shanghai Key Laboratory for Assisted Reproduction and Reproductive Genetics, Shanghai, China

**Keywords:** estradiol, cumulative live birth, freeze-all, embryo transfer, obstetric outcome

## Abstract

**Objective:**

To determine whether the peak serum estradiol (E2) level during ovarian stimulation affects the cumulative live birth rate (CLBR) and obstetric outcomes in freeze-all cycles.

**Methods:**

This retrospective cohort study involved patients who underwent their first cycle of *in vitro* fertilization followed by a freeze-all strategy and frozen embryo transfer cycles between January 2014 and June 2019 at a tertiary care center. Patients were categorized into four groups according to quartiles of peak serum E2 levels during ovarian stimulation (Q1-Q4). The primary outcome was CLBR. Secondary outcomes included obstetric and neonatal outcomes of singleton and twin pregnancies. Poisson or logistic regression was applied to control for potential confounders for outcome measures, as appropriate. Generalized estimating equations were used to account for multiple cycles from the same patient for the outcome of CLBR.

**Result(s):**

A total of 11237 patients were included in the analysis. Cumulatively, live births occurred in 8410 women (74.8%). The live birth rate (LBR) and CLBR improved as quartiles of peak E2 levels increased (49.7%, 52.1%, 54.9%, and 56.4% for LBR; 65.1%, 74.3%, 78.4%, and 81.6% for CLBR, from the lowest to the highest quartile of estradiol levels, respectively, *P*<0.001). Such association remained significant for CLBR after accounting for potential confounders in multivariable regression models, whereas the relationship between LBR and peak E2 levels did not reach statistical significance. In addition, no significant differences were noticed in adverse obstetric and neonatal outcomes (gestational diabetes mellitus, pregnancy-induced hypertension, preeclampsia, placental disorders, preterm birth, low birthweight, and small for gestational age) amongst E2 quartiles for either singleton or twin live births, both before and after adjustment.

**Conclusion:**

In freeze-all cycles, higher peak serum E2 levels during ovarian stimulation were associated with increased CLBR, without increasing the risks of adverse obstetric and neonatal outcomes.

## 1 Introduction

Controlled ovarian stimulation (COS) is undoubtedly one of the milestones in assisted reproductive treatments (1), which has resulted in a significant increase in pregnancy rates as compared with unstimulated *in vitro* fertilization (IVF) cycles (2, 3). However, COS, by stimulating multi-follicular growth, often increases serum estradiol (E2) to supraphysiologic levels, and the question of whether high E2 levels during COS may influence reproductive outcomes has been a matter of debate over the past few decades (4, 5). Existing data have reported that there may be a detrimental effect of high E2 level, which could lead to impaired endometrial receptivity (6–8). In addition, the increased incidence of ovarian hyperstimulation syndrome (OHSS) with high E2 exposure cannot be neglected (9). Studies have also suggested that a high response to ovarian stimulation may affect the quality of oocytes or embryos by altering the epigenetic programming of oocytes including DNA methylation, histone acetylation and epigenetic modifier expression (10–13), and potentially resulting in higher risks of implantation failure and pregnancy loss (14–16). Another concern is that the effect of supraphysiologic E2 level may further extend into placentation and subsequent fetal development, leading to higher risks of preeclampsia, low birthweight, and small for gestational age (SGA) (17–19).

Yet, published studies addressing the association between peak E2 level and pregnancy-related outcomes have focused mainly on fresh IVF cycles (4, 5), where top-quality embryos of the cohort were transferred into a suboptimal peri-implantation environment. In addition, very few data have reported the outcome of CLBR following multiple embryo transfer cycles after COS, which is of utmost importance to understand whether supraphysiologic E2 level during COS could affect the entire cohort of embryos. Taking into account the advances in cryopreservation technique, frozen embryo transfer (FET) has become an alternative to fresh embryo transfer (20), and FET cycles have contributed to an increased chance of live birth and better perinatal outcomes in clinical practice (21–23). Thus, it is vital to evaluate whether the high E2 levels during COS have any effects on CLBR and placentation following FET.

Given the increased utilization of the freeze-all strategy (24), which provides a novel model to assess separately the impact of ovarian stimulation on oocyte and embryo quality to that on the endometrium, we conducted the present study to investigate the association between peak serum E2 level during COS and CLBR, as well as obstetric and neonatal outcomes in freeze-all cycles.

## 2 Materials and methods

### 2.1 Study design

**Figure 1:**
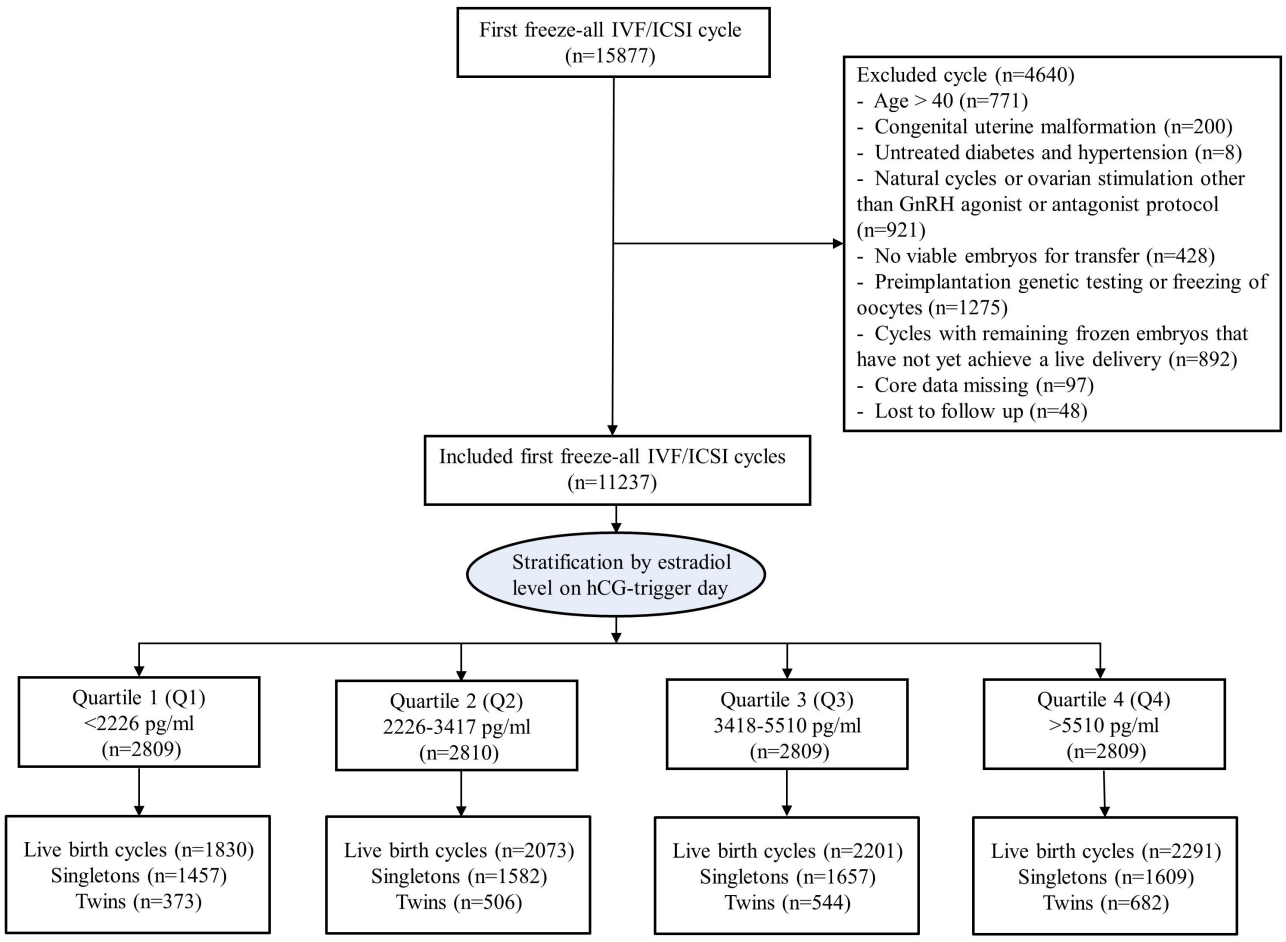
Flow chart.

This retrospective study was conducted at the Reproductive Center of Ren Ji Hospital of Shanghai Jiao Tong University School of Medicine. All patients aged 20-40 years old, undergoing their first autologous cycle of IVF or intracytoplasmic sperm injection (ICSI) treatment followed by a freeze-all strategy between January 2014 and June 2019 were reviewed for eligibility (**Figure 1**). Women who utilized gonadotropin-releasing hormone (GnRH) agonist or antagonist protocol for COS were included. Excluded were individuals diagnosed with congenital uterine malformation, or with untreated diabetes and hypertension, those with no viable embryos for transfer, and those undergoing preimplantation genetic testing or freezing of oocytes. Cycles with remaining frozen embryos that have not yet achieved a live delivery and those without available information on peak serum E2 level during COS and pregnancy outcomes were also excluded. The study protocol was approved by the Institutional Review Board of the hospital.

### 2.2 Ovarian stimulation protocols

Protocols for ovarian stimulation were determined at the discretion of patients’ preference and physicians’ recommendation. COS was performed with injections of 150-300 IU/day recombinant follicle-stimulating hormone (rFSH, Merck Serono) and/or urinary human menopausal gonadotropin (uHMG, Ferring). The starting dose was individualized based on the patient’s age, body mass index (BMI), and ovarian reserve makers. For patients using GnRH-agonist long protocol, Triptorelin (0.05 mg daily, Ferring) was administered on day 7 after ovulation and lasted for 10-14 days. For those using GnRH-agonist short protocol, Triptorelin (0.1 mg daily, Ferring) was injected starting on day 2 or 3 of their menstrual cycle and continued until the trigger day. In participants using the GnRH-antagonist protocol, the antagonist (0.25 mg by daily subcutaneous injection, Vetter Pharma-Fertigung GmbH & Co. KG or Merck Serono) was introduced when the leading follicle reached 12mm in average diameter.

Follicle development during COS was monitored by serial transvaginal ultrasound and serum E2, luteinizing hormone (LH), and progesterone (P) levels starting from day 4-5 of stimulation. Monitoring frequency was individualized, and the dose of gonadotropin (Gn) was adjusted accordingly. Final oocyte maturation was induced by administering 250 μg of recombinant human chorionic gonadotropin (hCG, Merck Serono) when at least one lead follicle reached 18 mm in mean diameter. Oocyte retrieval was conducted by vaginal ultrasound-guided puncture 36 hours later.

### 2.3 IVF, endometrial preparation, and embryo transfer

Retrieved oocytes were fertilized either via conventional IVF or ICSI based on serum analysis. Fertilization was examined 16-18 hours post insemination or microinjection by the presence of two pronuclei. Then the embryos were placed into individual droplets of cleavage culture medium (G1.5, Vitrolife, Gothenburg, Sweden) for three consecutive days and in the sequential culture medium (G2.5, Vitrolife, Gothenburg, Sweden) thereafter. Cleavage embryos with ≥6 blastomeres and <20% fragmentation on day 3 were defined as good quality and were frozen by vitrification. Embryos that did not meet the criteria were extendedly cultured for blastocyst, and those scored ≥4BC were eligible for vitrification on day 5 or 6 according to the Gardner criteria (25). Blastocysts scored ≥3BB were defined as good-quality embryos. Culture media, laboratory conditions, and procedures remain unchanged during the study period.

Endometrial preparation was performed in an artificial cycle, a modified natural cycle or a stimulated cycle. The endometrial preparation regimen was based on the physicians’ discretion. For the artificial cycle, oral administration of estrogen valerate (4-6 mg daily, Bayer Vital GmbH) was started on day 2-5 of the menstrual cycle, vaginal progesterone gel (90 mg daily; Merck Serono) and oral dydrogesterone (10 mg, 2-3 times daily; Abbott) were added when endometrial thickness reached 7 mm. For the modified natural cycle, ovulation was determined by serum hormone levels and ultrasound monitoring. For the stimulation cycle, letrozole (2.5 mg daily, Hengrui Pharma) was orally administered on cycle day 3 for 5 days, and follicle growth was monitored from cycle day 10. If the diameter of the dominant follicle was <14 mm, an additional 75 IU of uHMG was supplemented until the diameter ≥17 mm. If the diameter of the dominant follicle was ≥14 mm on cycle day 10, no more uHMG was given. In both the modified natural and stimulation cycles, ovulation was triggered by hCG either when the mean diameter of the dominant follicle was ≥17 mm or when the serum luteinizing hormone (LH) surge was detected, and oral dydrogesterone (10 mg, 2-3 times daily) was started 2 days after triggering for luteal phase support. In all FET cycles, no more than two embryos were transferred. Cleavage-stage embryos were transferred three days after progesterone administration and blastocysts were transferred five days after progesterone administration. If pregnancy was achieved, luteal support was continued to 10-12 weeks of gestation.

### 2.4 Outcome measures

The primary outcome of the study was CLBR. The secondary outcomes included obstetric and neonatal outcomes of live births, as well as pregnancy outcomes of the first FET cycle, namely the implantation rate, clinical pregnancy rate, early miscarriage rate, and live birth rate (LBR).

The implantation rate was defined as the number of fetal heartbeats observed per number of embryos transferred. Clinical pregnancy was defined as the observation of at least one gestational sac at 6-8 weeks of gestation. Early miscarriage rate was defined as a loss of clinical pregnancy before the 12th gestational week. Live birth was defined as the delivery of at least one living child (≥28 weeks of gestation). CLBR was calculated by including only the first live birth born after all FET cycles resulting from the associated ovarian stimulation.

Obstetric outcomes included gestational diabetes mellitus (GDM, 10th revision of the International Statistical Classification of Diseases and Related Health Problems [ICD-10] code O24.4), pregnancy-induced hypertension (PIH, ICD-10 code O13), preeclampsia (ICD-10 code O14-O15), and placental disorders (placenta previa [ICD-10 code O44], placental abruption [ICD-10 code O45], placenta accreta, placenta increta, or placenta percreta [ICD-10 code O43.21, O43.23]). Neonatal outcomes included gestational age, birthweight, preterm birth, low birthweight, SGA, and birth defects (ICD-10 codes Q00-Q99). Preterm birth was defined as delivery before 37 complete weeks of gestation. Low birthweight was defined as birthweight <2500g. SGA was defined as birthweight <10th percentile of gender-specific birthweight reference at the same gestational week (26). The dataset collected maternal and neonatal conditions from electronic medical records of neonates born in our university hospital. While for neonates delivered elsewhere, the information was obtained from responsible obstetricians and/or pediatricians at the local hospitals.

### 2.5 Statistical analysis

Patients were categorized into groups according to quartiles (Q1-Q4) of peak serum E2 levels on hCG-trigger day: Q1 (<2226 pg/ml), Q2 (2226-3417 pg/ml), Q3 (3418-5510 pg/ml) and Q4 (>5510 pg/ml). Descriptive statistics were presented as mean (standard deviation [SD]) or numbers and percentages according to the nature of the variables. The distribution of normality was tested by the Kolmogorov–Smirnov test, and nonparametric tests were preferred according to the results. The Mann-Whitney test was applied to analyze the between-group differences of continuous variables, while comparisons of categorical variables were performed by Pearson’s chi-squared test. Poisson regression was performed to investigate the effect of peak E2 level on implantation, and logistic regression was used to evaluate the impact of peak E2 level on clinical pregnancy, early miscarriage, live birth, and obstetric and neonatal outcomes. Multivariable generalized estimating equations (GEE) analysis was applied to fit the logistic regression models and further explored the possible relationship between E_2_ level and CLBR by accounting for the clustering of FET cycles within individuals. For pregnancy outcomes, confounding factors adjusted in the multivariable models included: maternal age, maternal BMI, primary or secondary infertility, parity, basal FSH, infertility diagnosis, protocol for stimulation, P level on hCG-trigger day, IVF or ICSI, endometrial preparation regimen, embryo developmental stage, embryo quality, and number of embryos transferred. For obstetric and neonatal outcomes, factors including maternal age, maternal BMI, primary or secondary infertility, parity, infertility diagnosis, protocol for stimulation, endometrial preparation regimen, embryo developmental stage, embryo quality, and number of embryos transferred were adjusted. The group of Q1 was taken as the reference group.

Additionally, the predictive probability of cumulative live birth according to E2 levels on hCG-trigger day and maternal age was evaluated using the generalized additive model (GAM). All statistical analyses were performed using R statistical programming language (version 4.2.1; R Foundation for Statistical Computing, Vienna, Austria). Two-tailed *P-*value <0.05 was considered statistically significant.

## 3 Results

### 3.1 Patient demographic and cycle characteristics

A total of 11237 women were included in the analysis, with an average age of 29.7 ± 3.8 years and a BMI of 21.8 ± 3.2 kg/m^2^. The mean ± SD peak serum E2 level on hCG-trigger day in the study cohort was 4065.7 ± 2456.6 pg/ml. The patients’ baseline and cycle characteristics are presented in **Table 1**. Women with peak E2 levels in the highest quartile (Q4) were younger, with lower BMI, and were more likely to be diagnosed with primary infertility and polycystic ovarian syndrome (PCOS). Regarding the outcomes of ovarian stimulation, women with higher E2 quartiles resulted in increased number of retrieved oocytes and good-quality embryos, while fertilization rates remained similar across groups. Furthermore, more subsequent FET cycles were observed in higher E2 quartiles, where more embryos were transferred cumulatively.

**Table 1:** Patient demographic and cycle characteristics.

Variable	Overall	Q1 (<2226 pg/ml)	Q2 (2226-3417 pg/ml)	Q3 (3418-5510 pg/ml)	Q4 (>5510 pg/ml)	*P* value
No. of patients	11237	2809	2810	2809	2809	
Maternal age (y)	29.7 ± 4.0	30.6 ± 4.2	29.8 ± 3.9	29.4 ± 3.8	29.0 ± 3.8	<0.001
Maternal BMI (kg/m^2^)	21.8 ± 3.2	22.6 ± 3.4	22.0 ± 3.1	21.6 ± 3.1	21.2 ± 2.8	<0.001
Infertility duration (y)	3.2 ± 2.3	3.2 ± 2.3	3.2 ± 2.3	3.3 ± 2.3	3.3 ± 2.2	0.035
Primary infertility	7629(67.9)	1811(64.5)	1867(66.4)	1956(69.6)	1995(71.0)	<0.001
Parity						<0.001
0	10407(92.6)	2560(91.1)	2574(91.6)	2623(93.4)	2650(94.3)	
≥1	830(7.4)	249(8.9)	236(8.4)	186(6.6)	159(5.7)	
Basal FSH (IU//L)	6.6 ± 1.7	6.9 ± 2.0	6.5 ± 1.7	6.5 ± 1.6	6.4 ± 1.6	<0.001
Infertility diagnosis						<0.001
Tubal	6103(54.3)	1541(54.9)	1526(54.3)	1536(54.7)	1500(53.4)	0.697
Diminished ovarian reserve	439(3.9)	336(12.0)	100(3.6)	3(0.1)	0(0)	<0.001
PCOS	2777(24.7)	546(19.4)	708(25.2)	726(25.8)	797(28.4)	<0.001
Endometriosis	874(7.8)	273(9.7)	244(8.7)	195(6.9)	162(5.8)	<0.001
Male factors	4021(35.8)	1036(36.9)	1015(36.1)	967(34.4)	1003(35.7)	0.275
Other	419(3.7)	103(3.7)	126(4.5)	117(4.2)	73(2.6)	0.001
Protocol for ovarian stimulation						<0.001
GnRH-agonist long	3887(34.6)	475 (16.9)	845(30.1)	1136(40.4)	1431(50.9)	
GnRH-agonist short	2802(24.9)	1047(37.3)	679(24.2)	561(20.0)	515(18.3)	
GnRH-antagonist	4548(40.5)	1287(45.8)	1286(45.8)	1112(39.6)	863(30.7)	
Total Gn dose (IU)	1453.8 ± 487.0	1494.7 ± 564.0	1457.7 ± 506.4	1457.4 ± 461.5	1405.3 ± 396.7	<0.001
Progesterone level on hCG-trigger day (ng/mL)	1.0 ± 0.7	0.7 ± 0.7	0.8 ± 0.5	1.0 ± 0.9	1.3 ± 0.7	<0.001
Cycles with ICSI	3199(28.5)	770(27.4)	813(28.9)	800(28.5)	816(29.0)	0.512
No. of oocytes retrieved	15.5 ± 7.7	9.4 ± 5.1	14.3 ± 5.7	17.4 ± 6.5	21.1 ± 8.1	<0.001
Fertilization rate	80.9 ± 16.0	80.6 ± 18.8	80.7 ± 16.6	80.9 ± 15.7	81.2 ± 15.5	0.458
No. of viable embryos	5.7 ± 3.6	3.7 ± 2.3	5.2 ± 2.9	6.3 ± 3.5	7.6 ± 4.1	<0.001
No. of good quality embryos	3.4 ± 2.5	2.1 ± 1.2	3.2 ± 2.3	3.6 ± 2.9	4.5 ± 3.4	<0.001
No. of FET cycles	1.5 ± 0.8	1.4 ± 0.6	1.5 ± 0.7	1.5 ± 0.8	1.6 ± 0.9	<0.001
No. of total embryos transferred	2.4 ± 1.3	2.1 ± 1.1	2.3 ± 1.3	2.5 ± 1.4	2.7 ± 1.5	<0.001
No. of cleavage-stage embryos transferred	1.7 ± 1.3	1.4 ± 1.1	1.6 ± 1.3	1.8 ± 1.3	2.0 ± 1.4	<0.001
No. of blastocysts transferred	0.7 ± 1.0	0.7 ± 0.9	0.8 ± 1.0	0.8 ± 1.1	0.7 ± 1.1	<0.001
FET endometrial preparation						<0.001
Artificial cycle	14006(84.0)	3294(85.5)	3507(83.5)	3616(84.2)	3589(82.5)	
Modified natural cycle	1398(8.4)	291(7.6)	332(7.9)	367(8.5)	408(9.4)	
Stimulated cycle	1227(7.7)	253(6.6)	362(8.6)	310(7.2)	352(8.1)	
Moderate or severe OHSS	59(0.5)	3(0.1)	7(0.2)	15(0.5)	34(1.2)	<0.001

Data are presented as mean ± standard deviation or number (%).

BMI, body mass index; FSH, follicle-stimulating hormone; PCOS, polycystic ovarian syndrome; GnRH, gonadotropin-releasing hormone; Gn, gonadotropin; IU, in units; hCG, human chorionic gonadotropin; ICSI, intracytoplasmic sperm injection; FET, Frozen embryo transfer; OHSS, ovarian hyperstimulation syndrome.

Moderate and severe OHSS occurred in 59 patients (0.5%) (27). The rates of OHSS were 0.1%, 0.2%, 0.5%, and 1.2% for Q1, Q2, Q3 and Q4 respectively, which increased significantly across groups (*P*<0.001).

### 3.2 Live birth rate and cumulative live birth rate

A total of 8410 (74.8%) women achieved live births following their FET cycles. Pregnancy outcomes of the first FET cycle and CLBR by quartiles of peak E2 levels are shown in **Table 2**. The clinical pregnancy rate, LBR, and CLBR improved as peak E2 quartiles increased (P<0.01), while the rate of implantation remained similar across different quartiles. The early miscarriage rate was lower in Q4 group compared with Q1 group in univariate analysis. After adjusting for potential confounders in multivariate regression models, results showed no statistically significant between peak E2 level and the rates of implantation, clinical pregnancy, early miscarriage, and live birth following the first FET. However, a positive association was detected between peak E2 level and CLBR after adjustment in multivariate regression and GEE models. The results of each FET cycle for the cumulative live birth are shown in **Supplemental Table 1**.

**Table 2:** Pregnancy outcomes and its association with peak serum estradiol levels.

Outcomes	Q1 (<2226 pg/ml)	Q2 (2226-3417 pg/ml)	Q3 (3418-5510 pg/ml)	Q4 (>5510 pg/ml)
Implantation rate ^a^				
n, (%)	2012/4352 (46.2)	2202/4594 (47.9)	2307/4787(48.2)	2425/5074(47.8)
Crude OR(95%CI) ^c^	Ref.	1.06(0.98-1.16)	1.04(0.95-1.14)	1.02(0.93-1.11)
Adjusted OR(95%CI) ^b,c^	Ref.	1.04(0.95-1.13)	1.04(0.94-1.14)	1.08(0.98-1.20)
Clinical pregnancy rate ^a^				
n, (%)	1685/2809(60.0)	1742/2810(62.0)	1796/2809(63.9)	1811/2809(64.5)
Crude OR(95%CI) ^d^	Ref.	1.09(0.98-1.22)	1.19(1.07-1.32) ^*^	1.21(1.09-1.35) ^*^
Adjusted OR(95%CI) ^b,d^	Ref.	0.98(0.88-1.10)	1.03(0.92-1.16)	1.08(0.95-1.22)
Early miscarriage rate ^a^				
n, (%)	216/1685(12.8)	211/1742(12.1)	183/1796(10.2)	174/1811(9.6)
Crude OR(95%CI) ^d^	Ref.	0.98(0.80-1.19)	0.84(0.68-1.03)	0.79(0.65-0.98) ^*^
Adjusted OR(95%CI) ^b,d^	Ref.	1.09(0.89-1.34)	1.01(0.81-1.27)	1.06(0.84-1.35)
LBR ^a^				
n, (%)	1397/2809(49.7)	1465/2810(52.1)	1541/2809(54.9)	1583/2809(56.4)
Crude OR(95%CI) ^d^	Ref.	1.10(0.99-1.22)	1.23(1.11-1.36) ^*^	1.31(1.18-1.45) ^*^
Adjusted OR(95%CI) ^b,d^	Ref.	0.97(0.87-1.08)	1.03(0.92-1.15)	1.08(0.96-1.22)
CLBR				
n, (%)	1830/2809(65.1)	2088/2810(74.3)	2201/2809(78.4)	2291/2809(81.6)
Crude OR(95%CI) ^d^	Ref.	1.55(1.38-1.74) ^*^	1.94(1.72-2.18) ^*^	2.37(2.09-2.68) ^*^
Adjusted OR(95%CI) ^b,e^	Ref.	1.06(0.96-1.16)	1.12(1.02-1.24) ^*^	1.21(1.09-1.35) ^*^

OR, odds ratio; CI, confidence interval; LBR, live birth rate; CLBR, cumulative live birth rate.

a Results of the first frozen embryo transfer cycle;

b Models were adjusted for maternal age, maternal BMI, primary or secondary infertility, parity, basal FSH, infertility diagnosis, protocol for stimulation, progesterone level on hCG day, IVF or ICSI, endometrial preparation regimen, embryo developmental stage, embryo quality, and number of embryos transferred; ^c^Results of poisson regression analysis; ^d^Results of logistic regression analysis; ^e^Results of generalized estimating equations regression analysis. *P value <0.05.

Analysis by age strata (<31, 31-34, 35-37, 38-40 years) showed a steady increase in CLBR with the peak E2 levels on hCG-trigger day (**Supplemental Figure 1**). However, for a given E2 level, CLBR decreased with increasing age, with the most prominent decline observed at 38-40 years old.

### 3.3 Maternal and neonatal outcomes

There were 6305 singletons (75.0%) and 2105 twins (25.0%) born during the study period (**Table 3**). No differences were noticed amongst peak E2 quartiles in terms of obstetric complications including GDM, PIH, preeclampsia, and placental disorders for both singleton and twin live births. Birthweights were similar amongst different quartiles. The incidence of preterm birth, low birthweight, SGA, and birth defect were also comparable across groups. Details of birth defects that occurred in all live-born babies were presented in **Supplemental Table 2**.

**Table 3:** Maternal complications and neonatal outcomes, stratified by estradiol levels on hCG-trigger day.

Outcome	Overall	Q1 (<2226 pg/ml)	Q2 (2226-3417 pg/ml)	Q3 (3418-5510 pg/ml)	Q4 (>5510 pg/ml)	*P* value
No. of live birth	8410	1830	2088	2201	2291	
Singleton	6305(75.0)	1457(79.6)	1582(75.8)	1657(75.3)	1609(70.2)	
Twins	2105(25.0)	373(20.4)	506(24.2)	544(24.7)	682(29.8)	
Gestational diabetes mellitus						
Singleton	754(12.0)	188(12.9)	202(12.8)	197(11.9)	167(10.4)	0.110
Twins	210(10.0)	43(11.5)	57(11.3)	55(10.1)	55(8.1)	0.193
Pregnancy-induced hypertension						
Singleton	262(4.2)	68(4.7)	71(4.5)	58(3.5)	65(4.0)	0.357
Twins	177(8.4)	35(9.4)	43 (8.5)	40(7.4)	59(8.7)	0.728
Preeclampsia						
Singleton	194(3.1)	52(3.6)	58(3.7)	44(2.7)	40(2.5)	0.117
Twins	136(6.5)	26(7.0)	37(7.3)	34(6.3)	39(5.7)	0.699
Placental disorders						
Singleton	146(2.3)	35(2.4)	41(2.6)	43(2.6)	27(1.7)	0.257
Twins	44(2.1)	8(2.1)	15(3.0)	11(2.0)	10(1.5)	0.361
Male gender						
Singleton	3354(53.2)	790(54.2)	822(52.0)	896(54.1)	846(52.6)	0.504
Twins	2188(52.0)	382(51.2)	537(53.1)	565(51.9)	704(51.6)	0.866
Gestational age (weeks)						
Singleton	38.7 ± 1.7	38.7 ± 1.7	38.7 ± 1.7	38.8 ± 1.6	38.8 ± 1.8	0.134
Twins	36.0 ± 1.9	35.9 ± 2.1	35.9 ± 1.0	36.2 ± 1.8	36.1 ± 1.9	0.083
Preterm birth						
Singleton	422(6.7)	96(6.6)	126(8.0)	94(5.7)	106(6.6)	0.075
Twins	1021(48.5)	182(48.8)	258(51.0)	251(46.1)	330(48.4)	0.478
Birthweight (g)						
Singleton	3388.0 ± 513.8	3406.0 ± 527.3	3374.4 ± 520.2	3404.7 ± 493.3	3367.7 ± 515.3	0.066
Twins	2552.4 ± 426.2	2539.3 ± 466.5	2538.9 ± 434.1	2571.6 ± 398.9	2554.3 ± 430.4	0.578
Low birthweight						
Singleton	264(4.2)	61(4.2)	69(4.4)	64(3.9)	70(4.4)	0.882
Twins	1593(37.8)	282(37.8)	388(38.3)	392(36.0)	531(38.9)	0.510
SGA						
Singleton	286(4.5)	65(4.5)	74(4.7)	66(4.0)	81(5.0)	0.535
Twins	149(3.5)	34(4.6)	34(3.4)	31(2.8)	50(3.7)	0.268
Birth defect						
Singleton	175(1.3)	17(1.2)	17(1.1)	22(1.3)	24(1.5)	0.734
Twins	66(1.6)	11(1.5)	18(1.8)	15(1.4)	22(1.6)	0.896

Data are presented as mean ± standard deviation or number (percentage).

SGA, small for gestational age.

**Figure 2:**
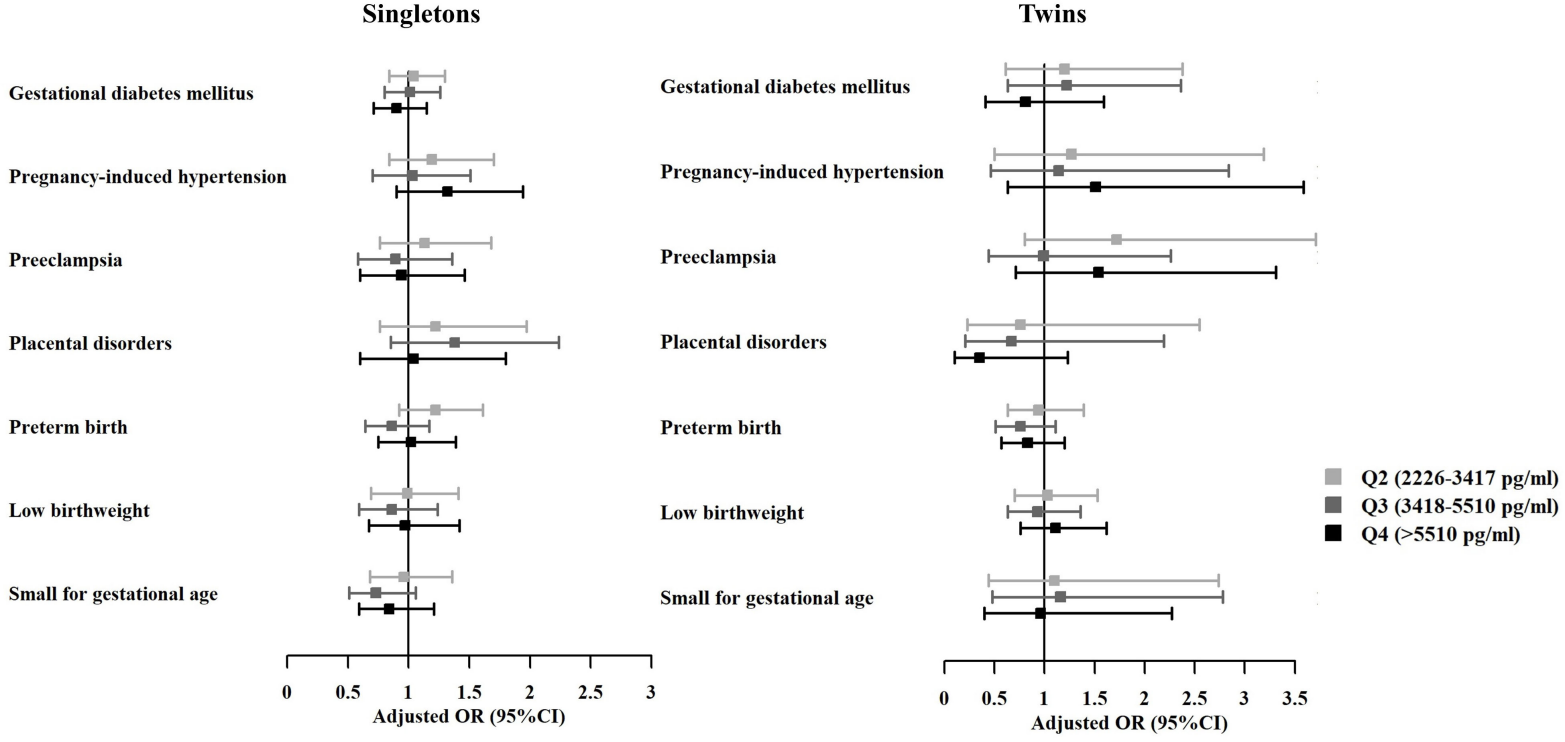
Adjusted odds ratios of adverse maternal and neonatal outcomes among live births with different estradiol levels on hCG-trigger day. OR, odds ratio; CI, confidence interval. The analyses were adjusted for maternal age, maternal BMI, primary or secondary infertility, parity, infertility diagnosis, protocol for stimulation, endometrial preparation regimen, embryo developmental stage, embryo quality, and number of embryos transferred (The group of Q1 was taken as the reference group).

Results of multivariable logistic regression adjusting for potential confounders revealed no associations between peak E2 level and adverse obstetric and neonatal outcomes (**Figure 2**).

## 4 Discussion

Results of this large cohort study demonstrated that peak serum E2 level during COS was positively associated with CLBR in freeze-all cycles, while no association was found between E2 level and adverse obstetric and neonatal outcomes.

In fresh embryo transfer cycles, evidence about whether the peak serum E2 level during COS affects pregnancy outcomes remains conflicting (4, 5). The heterogeneity of the population sampled, study sizes and E2 cut-off levels in these studies may account for their discrepancies. For instance, Moralog˘lu et al. involved 106 patients with ≥ 5 oocytes retrieved and suggested that peak E2 levels >2500 pg/ml were negatively associated with implantation rates (16). However, a large cohort study by Mustafa et al. included 6478 ICSI cycles and found that E2 levels over the 90^th^ percentile (>4200 pg/ml) had increased clinical pregnancy rate, while the implantation rate was similar across the E2 percentile groups (28). Bianco et al. using a threshold of 2000 pg/ml, reviewed 58 oocyte donation cycles and reported that E2 concentration did not affect clinical pregnancy rate and LBR (29). In addition, other researchers showed no influence of peak E2 levels during COS on IVF success rates in autologous cycles (30–33). Among the existing studies, Yu Ng et al. and Chen et al. have further explored the subsequent FET cycles after the initial fresh cycles and reported similar pregnancy outcomes across different E2 concentrations, but they mainly looked at the rates of implantation and clinical pregnancy, without evaluating the CLBR (31, 32).

The impact of peak E2 levels during COS on the CLBR can be interpreted as a useful indicator of its effect on embryo development and implantation potential. A retrospective study included 1141 non-PCOS patients and assessed the outcomes of fresh and frozen cycles (34). They reported that the peak E2 level had a concentration-dependent effect on CLBR, with the optimal CLBR achieved between the E2 range of 2185-6361 pg/ml and a remarkable decrease afterward. However, the results could have been confounded since the CLBR in this study was calculated based on pooled data of both fresh and frozen cycles in the first embryo transfer attempt. Besides, the authors acknowledged that the sample size was relatively small in high E2 levels. The present study, conducted in a large general population, has estimated both reproductive outcomes after the first FET cycle and CLBR to explore the applicability of the freeze-all strategy in IVF patients with different peak E2 levels. Our results found that a higher E2 level not only does not decrease the implantation rate and LBR but, on the contrary, increases CLBR following the use of frozen-thawed embryos. Furthermore, the benefit from high ovarian response is limited for patients with advanced age as the CLBR reaches a plateau in extremely elevated E2 levels. These results, based on the freeze-all setting, added information on the association between peak E2 levels during COS and CLBR.

Concerning obstetric and neonatal outcomes, the maternal hyperestrogenic milieu has been reported to has an adverse effect on placentation and subsequent fetal growth (17–19). In comparison with previous studies presenting increased risks of preeclampsia, low birthweight and SGA with elevating E2 levels in fresh embryo transfer cycles, the present study found no association between E2 levels and adverse maternal and neonatal outcomes. Cai et al. and Zhang et al. conducted their studies in FET cycles and suggested that singleton birthweight was negatively influenced by increasing peak E2 levels during COS (35, 36). However, both studies were limited by the main FET indication of failed fresh transfer and OHSS risk, which would lead to confounding outcomes as the patients included were generally with worse prognoses. Consistent with our findings, a more recent large cohort study reported that peak E2 level during COS was not related to increased risks of low birthweight and SGA in freeze-all cycles, although patients with maternal complications including GDM and hypertensive disorders were excluded in the study (37).

A significant body of evidence has demonstrated that the supraphysiologic E2 level in IVF treatments may impair endometrial receptivity and adversely affect trophoblastic invasion or placentation, which may explain the unfavorable results associated with high E2 levels in fresh cycles (6–8, 17–19). However, it is also of concern whether there is a negative effect on the quality of oocytes or embryos attributable to high E2 levels (10–13). Many studies indicated that the embryonic viability decreased and chromosomal abnormalities increased after superovulation in animal experiments (10, 11). In addition, hormonal stimulation has been hypothesized to induce epigenetic alterations in both human and murine oocytes or embryos derived from assisted reproduction treatment (12, 13). However, contemporary studies have reported that ovarian stimulation was not related to the chromosomal status of embryos (38, 39) and no drastic epigenetic changes were found in placental tissues with or without superovulation (40).

Furthermore, it is difficult to distinguish the effects of supraphysiologic E2 level on oocytes or embryos from those on the endometrium in fresh cycles, whereas a freeze-all strategy provides a novel model to assess the sole impact of ovarian stimulation on oocyte and embryo quality after ruling out the potential deleterious influences on endometrium caused by a hyperestrogenic milieu (41). Our study, focusing on pregnancy and obstetric outcomes, adds further to the currently existing evidence by suggesting that the high E2 levels do not appear to pose adverse effects on oocyte or embryo quality, and the detrimental effect of intrauterine high E2 exposure could be avoided by transferring embryos into a more physiologic uterine environment.

This is the first study to evaluate the impact of peak E2 level during COS in freeze-all cycles. The strength of our study is the large cohort size with an organized dataset that offered all the relevant parameters in the analysis. The primary outcome of this study, CLBR, allows us to capture all live births after one ovarian stimulation cycle and the corresponding obstetric and fetal outcomes, which also provides new insight into the relationship between E2 level and the success of an IVF program.

We acknowledged that there are limitations in this study. The retrospective nature of the analysis may increase the chance of selection bias regarding the population characteristics as well as cycle parameters (e.g. basal ovarian reserve, fertilization method, embryo stage at transfer). In this regard, we utilized multivariable regression models to adjust for potential confounders, and the result of CLBR was reinforced by the GEE analysis. In addition, the policy of transferring two cleavage-stage embryos was taken as a priority in our IVF centers before 2019 and single blastocyst transfer was encouraged afterward given the advantages of reduced multiple pregnancies and improved pregnancy rates (42, 43). Thus, the results of our analysis may not be generalizable to other populations where blastocysts were cultured and transferred primarily. Further investigations on this subject are still needed to evaluate the effect of high E2 levels on the oocyte competence and embryo developmental potential, as well as the long-term health of IVF offspring.

## 5 Conclusion

Our study demonstrated that, in freeze-all cycles, the CLBR progressively increased with the higher levels of peak serum E2 after COS, while the risks of adverse obstetric and neonatal outcomes were not increased, suggesting that high E2 levels may actually have a very limited or no adverse effect on oocyte or embryo quality. Our results provide reassuring findings for patients with high E2 levels during COS and suggest that they may benefit from freeze-all cycles. Nevertheless, given that the extremely elevated E2 levels would pose additional risks such as OHSS and thromboembolic complications after oocyte retrieval, COS for freeze-all cycles should be rational to avoid aggressive stimulation and focus on the balance between treatment efficiency and patients’ safety.

## Data availability statement

The raw data supporting the conclusions of this article will be made available by the authors, without undue reservation.

## Ethics statement

The studies involving human participants were reviewed and approved by The Ethics Committee of Ren Ji Hospital, School of Medicine, Shanghai Jiao Tong University. This study did not require informed consent for participation following the national legislation and the institutional requirements.

## Author contributions

JH and YL: study design, analysis and interpretation of data, and drafting of the manuscript. YW, QZ, JQ, YD and HZ: data collection. YH and ZD: assessed the article. YS: study concept and revise of article. All authors contributed to the article and approved the submitted version.

## References

[1] Trounson AO Leeton JF Wood C Webb J Wood J. Pregnancies in humans by fertilization *in vitro* and embryo transfer in the controlled ovulatory cycle. Science (1981) 212(4495):681–2. doi: 10.1126/science.7221557 7221556

[2] Edwards RG Steptoe PC Purdy JM. Establishing full-term human pregnancies using cleaving embryos grown *in vitro* . Br J Obstet Gynaecol (1980) 87(9):737–56. doi: 10.1111/j.1471-0528.1980.tb04610.x 6775685

[3] Macklon NS Stouffer RL Giudice LC Fauser BC. The science behind 25 years of ovarian stimulation for *in vitro* fertilization. Endocr Rev (2006) 27(2):170–207. doi: 10.1210/er.2005-0015 16434510

[4] Kosmas IP Kolibianakis EM Devroey P. Association of estradiol levels on the day of hCG administration and pregnancy achievement in IVF: a systematic review. Hum Reprod (2004) 19(11):2446–53. doi: 10.1093/humrep/deh473 15471938

[5] Karatasiou GI Bosdou JK Venetis CA Zepiridis L Chatzimeletiou K Tarlatzi TB. Is the probability of pregnancy after ovarian stimulation for IVF associated with serum estradiol levels on the day of triggering final oocyte maturation with hCG? A systematic review and meta-analysis. J Assist Reprod Genet (2020) 37(7):1531–41. doi: 10.1007/s10815-020-01829-z PMC7376780 32472447

[6] Kolibianakis E Bourgain C Albano C Osmanagaoglu K Smitz J Steirteghem AV. Effect of ovarian stimulation with recombinant follicle-stimulating hormone, gonadotropin releasing hormone antagonists, and human chorionic gonadotropin on endometrial maturation on the day of oocyte pick-up. Fertil Steril (2002) 78(5):1025–9. doi: 10.1016/s0015-0282(02)03323-x 12413988

[7] Ng EH Chan CC Tang OS Yeung WS Ho PC. Comparison of endometrial and subendometrial blood flow measured by three-dimensional power Doppler ultrasound between stimulated and natural cycles in the same patients. Hum Reprod (2004) 19(10):2385–90. doi: 10.1093/humrep/deh384 15319389

[8] Haouzi D Assou S Mahmoud K Tondeur S Reme T Hedon B. Gene expression profile of human endometrial receptivity: comparison between natural and stimulated cycles for the same patients. Hum Reprod (2009) 24(6):1436–45. doi: 10.1093/humrep/dep039 PMC2871799 19246470

[9] Ng EH Lau EY Yeung WS Ho PC. Oocyte and embryo quality in patients with excessive ovarian response during *in vitro* fertilization treatment. J Assist Reprod Genet (2003) 20(5):186–91. doi: 10.1023/a:1023670010031 PMC3455303 12812461

[10] Van der Auwera I D’Hooghe T. Superovulation of female mice delays embryonic and fetal development. Hum Reprod (2001) 16(6):1237–43. doi: 10.1093/humrep/16.6.1237 11387298

[11] Roberts R Iatropoulou A Ciantar D Stark J Becker DL Franks S. Follicle-stimulating hormone affects metaphase I chromosome alignment and increases aneuploidy in mouse oocytes matured in Vitro1. Biol Reprod (2005) 72(1):107–18. doi: 10.1095/biolreprod.104.032003 15371272

[12] Sato A Otsu E Negishi H Utsunomiya T Arima T. Aberrant DNA methylation of imprinted loci in superovulated oocytes. Hum Reprod (2007) 22(1):26–35. doi: 10.1093/humrep/del316 16923747

[13] Market-Velker BA Zhang L Magri LS Bonvissuto AC Mann MR. Dual effects of superovulation: loss of maternal and paternal imprinted methylation in a dose-dependent manner. Hum Mol Genet (2010) 19(1):36–51. doi: 10.1093/hmg/ddp465 19805400

[14] Valbuena D Martin J de Pablo JL Remohı´ J Pellicer A Simo´n C. Increasing levels of estradiol are deleterious to embryonic implantation because they directly affect the embryo. Fertil Steril (2001) 76(5):962–8. doi: 10.1016/s0015-0282(01)02018-0 11704118

[15] Wang JX Norman RJ Wilcox AJ. Incidence of spontaneous abortion among pregnancies produced by assisted reproductive technology. Hum Reprod (2004) 19(2):272–7. doi: 10.1093/humrep/deh078 14747166

[16] Moraloglu O Tonguc EA Ozel M Ozaksit G Var T Sarikaya E. The effects of peak and mid-luteal estradiol levels on *in vitro* fertilization outcome. Arch Gynecol Obstet (2012) 285(3):857–62. doi: 10.1007/s00404-011-2090-8 21938500

[17] Farhi J Ben-Haroush A Andrawus N Pinkas H Sapir O Fisch B. High serum oestradiol concentrations in IVF cycles increase the risk of pregnancy complications related to abnormal placentation. Reprod BioMed Online (2010) 21(3):331–7. doi: 10.1016/j.rbmo.2010.04.022 20688571

[18] Imudia AN Awonuga AO Doyle JO Kaimal AJ Wright DL Toth TL. Peak serum estradiol level during controlled ovarian hyperstimulation is associated with increased risk of small for gestational age and preeclampsia in singleton pregnancies after *in vitro* fertilization. Fertil Steril (2012) 97(6):1374–9. doi: 10.1016/j.fertnstert.2012.03.028 22494926

[19] Pereira N Elias RT Christos PJ Petrini AC Hancock K Lekovich JP. Supraphysiologic estradiol is an independent predictor of low birth weight in full-term singletons born after fresh embryo transfer. Hum Reprod (2017) 32(7):1410–7. doi: 10.1093/humrep/dex095 PMC6251696 28505290

[20] Barnhart KT. Introduction: are we ready to eliminate the transfer of fresh embryos in *in vitro* fertilization? Fertil Steril (2014) 102(1):1–2. doi: 10.1016/j.fertnstert.2014.05.024 24890272 PMC4146486

[21] Zaat T Zagers M Mol F Goddijn M van Wely M Mastenbroek S. Fresh versus frozen embryo transfers in assisted reproduction. Cochrane Database Syst Rev (2021) 2(2):2CD011184. doi: 10.1002/14651858.CD011184.pub3 PMC8095009 33539543

[22] Jarvela IY Pelkonen S Uimari O Makikallio K Puukka K Ruokonen A. Controlled ovarian hyperstimulation leads to high progesterone and estradiol levels during early pregnancy. Hum Reprod (2014) 29(11):2393–401. doi: 10.1093/humrep/deu223 25205752

[23] Sha T Yin X Cheng W Massey IY. Pregnancy-related complications and perinatal outcomes resulting from transfer of cryopreserved versus fresh embryos *in vitro* fertilization: a meta-analysis. Fertil Steril (2018) 109(2):330–42. doi: 10.1016/j.fertnstert.2017.10.019 29331236

[24] Blockeel C Drakopoulos P Santos-Ribeiro S Polyzos NP Tournaye H. A fresh look at the freeze-all protocol: a SWOT analysis. Hum Reprod (2016) 31(3):491–7. doi: 10.1093/humrep/dev339 26724793

[25] Gardner DK Lane M Stevens J Schlenker T Schoolcraft WB. Blastocyst score affects implantation and pregnancy outcome: towards a single blastocyst transfer. Fertil Steril (2000) 73(6):1155–8. doi: 10.1016/s0015-0282(00)00518-5 10856474

[26] Dai L Deng C Li Y Zhu J Mu Y Deng Y. Birth weight reference percentiles for Chinese. PloS One (2014) 9(8):e104779. doi: 10.1371/journal.pone.0104779 25127131 PMC4134219

[27] Golan A Ron-el R Herman A Soffer Y Weinraub Z Caspi E. Ovarian hyper- stimulation syndrome: an update review. Obstet Gynecol Surv (1989) 44(6):430–40. doi: 10.1097/00006254-198906000-00004 2660037

[28] Bahçeci M Ulug U Erden HF Mesut A Jozwiak EA. Elevated oestradiol concentrations are not associated with increased first trimester miscarriage rates of singleton gestations conceived by assisted conception treatment. Reprod BioMed Online (2006) 12(1):33–8. doi: 10.1016/s1472-6483(10)60977-7 16454931

[29] Bianco K Mahutte NG Arici A Sakkas D Taylor HS. Effect of estradiol on oocyte development. Int J Gynaecol Obstet (2009) 104(3):230–2. doi: 10.1016/j.ijgo.2008.10.015 PMC3107851 19056082

[30] Sharara FI McClamrock HD. High estradiol levels and high oocyte yield are not detrimental to *in vitro* fertilization outcome. Fertil Steril (1999) 72(3):401–5. doi: 10.1016/S0015-0282(99)00293-9 10519607

[31] Yu Ng EH Yeung WS Yee Lan Lau E So WW Ho PC. High serum E2 concentrations in fresh IVF cycles do not impair implantation and pregnancy rates in subsequent frozen-thawed embryo transfer cycles. Hum Reprod (2000) 15(2):250–5. doi: 10.1093/humrep/15.2.250 10655293

[32] Chen QJ Sun XX Li L Gao XH Wu Y Gemzell-Danielsson K. Effects of ovarian high response on implantation and pregnancy outcome during controlled ovarian hyperstimulation (with GnRH agonist and rFSH). Acta Obstet Gynecol Scand (2007) 86(7):849–54. doi: 10.1080/00016340701415152 17611831

[33] Kyrou D Popovic-Todorovic B Fatemi HM Bourgain C Haentjens P Van Landuyt L. Does the estradiol level on the day of human chorionic gonadotrophin administration have an impact on pregnancy rates in patients treated with rec-FSH/GnRH antagonist? Hum Reprod (2009) 24(11):2902–9. doi: 10.1093/humrep/dep290 19671625

[34] Zhang W Tian Y Xie D Miao Y Liu J Wang X. The impact of peak estradiol during controlled ovarian stimulation on the cumulative live birth rate of IVF/ICSI in non-PCOS patients. J Assist Reprod Genet (2019) 36(11):2333–44. doi: 10.1007/s10815-019-01568-w PMC6885456 31485870

[35] Zhang W Ma Y Xiong Y Xiao X Chen S Wang X. Supraphysiological serum oestradiol negatively affects birthweight in cryopreserved embryo transfers: a retrospective cohort study. Reprod BioMed Online (2019) 39(2):312–20. doi: 10.1016/j.rbmo.2019.04.015 31255605

[36] Cai J Liu L Xu Y Liu Z Jiang X Li P. Supraphysiological estradiol level in ovarian stimulation cycles affects the birthweight of neonates conceived through subsequent frozen-thawed cycles: a retrospective study. BJOG (2019) 126(6):711–8. doi: 10.1111/1471-0528.15606 30628169

[37] Kuang Y Cai R Lyu Q Gao H Chen Q Lin J. Association between peak serum estradiol level during controlled ovarian stimulation and neonatal birthweight in freeze-all cycles: a retrospective study of 8501 singleton live births. Hum Reprod (2020) 35(2):424–33. doi: 10.1093/humrep/dez262 32078675

[38] Anckaert E Adriaenssens T Romero S Dremier S Smitz J. Unaltered imprinting establishment of key imprinted genes in mouse oocytes after *in vitro* follicle culture under variable follicle-stimulating hormone exposure. Int J Dev Biol (2009) 53(4):541–8. doi: 10.1387/ijdb.082619ea 19247969

[39] Irani M Canon C Robles A Maddy B Gunnala V Qin X. No effect of ovarian stimulation and oocyte yield on euploidy and live birth rates: an analysis of 12 298 trophectoderm biopsies. Hum Reprod (2020) 35(5):1082–9. doi: 10.1093/humrep/deaa028 32348476

[40] de Waal E Vrooman LA Fischer E Ord T Mainigi MA Coutifaris C. The cumulative effect of assisted reproduction procedures on placental development and epigenetic perturbations in a mouse model. Hum Mol Genet (2015) 24(24):6975–85. doi: 10.1093/hmg/ddv400 PMC4654053 26401051

[41] Shapiro BS Daneshmand ST Garner FC Aguirre M Hudson C Thomas S. Evidence of impaired endometrial receptivity after ovarian stimulation for *in vitro* fertilization: a prospective randomized trial comparing fresh and frozen-thawed embryo transfers in high responders. Fertil Steril (2011) 96(2):516–8. doi: 10.1016/j.fertnstert.2011.02.059 21737071

[42] Shi Y Sun Y Hao C Zhang H Wei D Zhang Y. Transfer of fresh versus frozen embryos in ovulatory women. N Engl J Med (2018) 378(2):126–36. doi: 10.1056/NEJMoa1705334 29320646

[43] Wei D Liu J-Y Sun Y Shi Y Zhang B Liu J-Q. Frozen versus fresh single blastocyst transfer in ovulatory women: a multicentre, randomised controlled trial. Lancet (2019) 393(10178):1310–8. doi: 10.1016/s0140-6736(18)32843-5 30827784

