# Supplementary figures and images for: The effect of peak serum estradiol level during ovarian stimulation on cumulative live birth and obstetric outcomes in freeze-all cycles
Source: Front Endocrinol (Lausanne). 2023 Jul 17;14:1130211. doi: 10.3389/fendo.2023.1130211 (PMC10390295; doi:10.3389/fendo.2023.1130211)

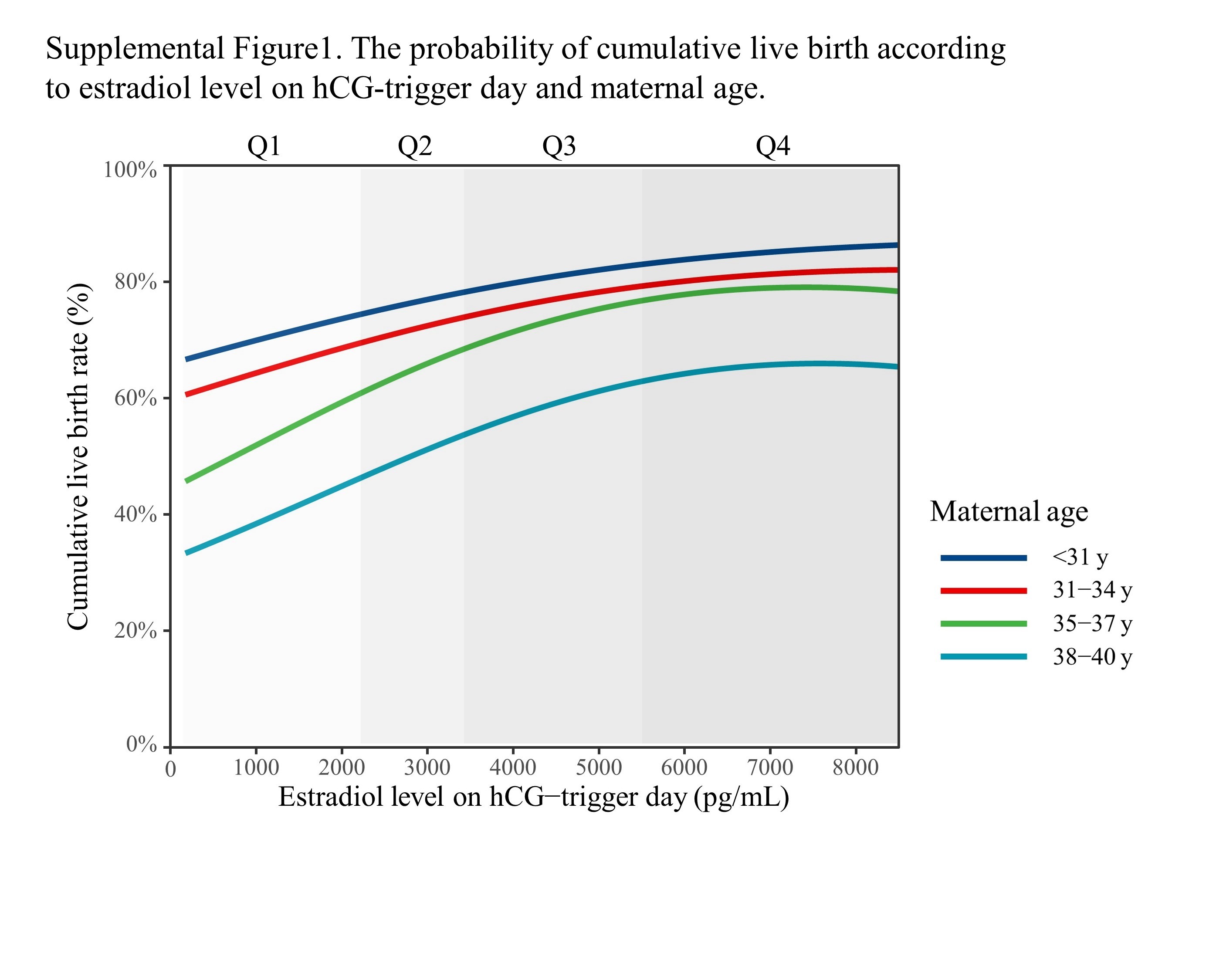

Supplement: Supplementary file 1 [file Image_1.jpeg]
