# Supplementary material for: The effect of peak serum estradiol level during ovarian stimulation on cumulative live birth and obstetric outcomes in freeze-all cycles
Source: Front Endocrinol (Lausanne). 2023 Jul 17;14:1130211. doi: 10.3389/fendo.2023.1130211 (PMC10390295; doi:10.3389/fendo.2023.1130211)
Supplement: Supplementary file 2 [file Table_1.docx]

Supplemental Table 1. Cycle results for the cumulative live birth according to estradiol levels on hCG-trigger day**.**

|  | Q1  (<2226 pg/ml) | Q2  (2226-3417 pg/ml) | Q3  (3418-5510 pg/ml) | Q4  (>5510 pg/ml) |
| --- | --- | --- | --- | --- |
| Cycle 1 |  |  |  |  |
| Implantation rate | 2052/4352 (47.2) | 2202/4594 (47.9) | 2307/4787(48.2) | 2425/5074(47.8) |
| Clinical pregnancy rate | 1685/2809(60.0) | 1742/2810(62.0) | 1796/2809(63.9) | 1811/2809(64.5) |
| Early miscarriage rate | 216/1685(12.8) | 211/1742(12.1) | 183/1796(10.2) | 174/1811(9.6) |
| LBR | 1397/2809(49.7) | 1465/2810(52.1) | 1541/2809(54.9) | 1583/2809(56.4) |
| Cycle 2 |  |  |  |  |
| Implantation rate | 512/1141(44.9) | 652/1465(44.5) | 668/1572(42.5) | 724/1711(42.3) |
| Clinical pregnancy rate | 431/806(53.5) | 546/996(54.8) | 558/1019(54.8) | 571/1033(55.3) |
| Early miscarriage rate | 67/431(15.5) | 70/546(12.8) | 69/558(12.4) | 64/571(11.2) |
| LBR | 344/806(42.7) | 461/996(46.3) | 469/1019(46.0) | 486/1033(47.0) |
| CLBR | 1741/2809(62.0) | 1926/2810(68.5) | 2010/2809(71.6) | 2069/2809(73.7) |
| Cycle 3 |  |  |  |  |
| Implantation rate | 113/265(42.6) | 192/452(42.5) | 225/530(42.5) | 236/578(40.8) |
| Clinical pregnancy rate | 100/197(50.8) | 171/331(51.7) | 186/352(52.8) | 186/366(50.8) |
| Early miscarriage rate | 13/100(13.0) | 26/171(15.2) | 23/186(12.4) | 18/186(9.7) |
| LBR | 80/197(40.6) | 143/331(43.2) | 155/352(44.0) | 162/366(44.3) |
| CLBR | 1821/2809(64.8) | 2069/2810(73.6) | 2165/2809(77.1) | 2231/2809(79.4) |
| Cycle 4 |  |  |  |  |
| Implantation rate | 13/35(37.1) | 28/77(36.4) | 51/127(40.2) | 58/165(35.2) |
| Clinical pregnancy rate | 12/25(48.0) | 23/54(42.6) | 42/86(48.8) | 50/106(47.2) |
| Early miscarriage rate | 2/12(16.7) | 2/23(8.7) | 8/43(18.6) | 4/50(8.0) |
| LBR | 9/25(36.0) | 18/54(33.3) | 30/86(34.9) | 44/106(41.5) |
| CLBR | 1830/2809(65.1) | 2087/2810(74.3) | 2195/2809(78.1) | 2275/2809(81.0) |
| Cycle 5 |  |  |  |  |
| Implantation rate | 0/2(0) | 1/18(5.6) | 2/32(6.3) | 23/49(46.9) |
| Clinical pregnancy rate | 0/1(0) | 1/10(10.0) | 2/19(10.5) | 19/30(63.0) |
| Early miscarriage rate | 0 | 0/1(0) | 0/2(0) | 4/19(21.1) |
| LBR | 0/1(0) | 1/10(10.0) | 2/19(10.5) | 15/30(50.0) |
| CLBR | 1830/2809(65.1) | 2088/2810(74.3) | 2197/2809(78.2) | 2290/2809(81.5) |
| Cycle 6 |  |  |  |  |
| Implantation rate | 0 | 0 | 4/13(30.8) | 1/9(11.1) |
| Clinical pregnancy rate | 0 | 0 | 4/7(57.1) | 1/5(20.0) |
| Early miscarriage rate | 0 | 0 | 0/4(0) | 0/1(0) |
| LBR | 0 | 0 | 4/7(57.1) | 1/5(20.0) |
| CLBR | 1830/2809(65.1) | 2088/2810(74.3) | 2201/2809(78.4) | 2291/2809(81.6) |

*Note:* Data are presented as number (%)

LBR = live birth rate; CLBR = cumulative live birth rate.

*Number of women not having a live birth and not continuing transfer cycles with left embryos, data are presented as number (% / number of no live birth)
