# Supplementary material for: The effect of peak serum estradiol level during ovarian stimulation on cumulative live birth and obstetric outcomes in freeze-all cycles
Source: Front Endocrinol (Lausanne). 2023 Jul 17;14:1130211. doi: 10.3389/fendo.2023.1130211 (PMC10390295; doi:10.3389/fendo.2023.1130211)
Supplement: Supplementary file 3 [file Table_2.docx]

Supplemental Table 2. Number of birth defects by estradiol levels on hCG-trigger day.

| Defect (International Classification of Diseases Code) | Overall | Q1  (<2226 pg/ml) | Q2  (2226-3417 pg/ml) | Q3  (3418-5510 pg/ml) | Q4  (>5510 pg/ml) |
| --- | --- | --- | --- | --- | --- |
| No. of live births | 8410 | 1830 | 2088 | 2201 | 2291 |
| Any defect | 146 | 28 | 35 | 37 | 46 |
| Multiple defects | 5 | 2 | 1 | 0 | 2 |
| Nervous system (Q00–Q07) | 4 | 1 | 0 | 1 | 2 |
| Eye, ear, face and neck (Q10–Q18) | 19 | 3 | 4 | 6 | 6 |
| Circulatory system (Q20–Q28) | 67 | 14 | 15 | 17 | 21 |
| Respiratory system (Q30–Q34) | 4 | 1 | 2 | 0 | 1 |
| Cleft lip and cleft palate (Q35–Q37) | 4 | 0 | 1 | 1 | 2 |
| Digestive system (Q38–Q45) | 10 | 1 | 4 | 3 | 2 |
| Genital organs (Q50–Q56) | 2 | 0 | 1 | 0 | 1 |
| Urinary system (Q60–Q64) | 8 | 2 | 2 | 1 | 3 |
| Musculoskeletal system (Q65–Q79) | 27 | 6 | 6 | 8 | 7 |
| Other (Q80–Q89) | 4 | 1 | 1 | 0 | 2 |
| Chromosomal abnormalities (Q90–Q99) | 2 | 1 | 0 | 0 | 1 |
